# Comprehensive geriatric assessment predicts listing for kidney transplant in patients with end-stage renal disease: a retrospective cohort study

**DOI:** 10.1186/s12877-024-04734-7

**Published:** 2024-02-13

**Authors:** Jay Patel, Michelle Martinchek, Dawson Mills, Sheraz Hussain, Yousef Kyeso, Megan Huisingh-Scheetz, Daniel Rubin, Andrea J. Landi, Arielle Cimeno, Maria Lucia L. Madariaga

**Affiliations:** 1 Pritzker School of Medicine, University of Chicago, 5841 S. Maryland Ave. MC5047, 60637 Chicago, IL USA; 2 Geriatrics and Extended Care and New England Geriatrics Research Education and Clinical Center, Veterans Affairs Boston Healthcare System, Boston, MA USA; 3 Department of Medicine, University of Chicago Medicine & Biological Sciences, Chicago, USA; 4 Department of Anesthesia, University of Chicago Medicine & Biological Sciences, Chicago, USA; 5 Department of Surgery, University of Chicago Medicine & Biological Sciences, Chicago, USA

**Keywords:** End-stage renal disease, Comprehensive geriatric assessment, Frailty, Kidney transplant waitlist, Older adults

## Abstract

**Background:**

Comprehensive geriatric assessment (CGA) involves a formal broad approach to assess frailty and creating a plan for management. However, the impact of CGA and its components on listing for kidney transplant in older adults has not been investigated.

**Methods:**

We performed a single-center retrospective study of patients with end-stage renal disease who underwent CGA during kidney transplant candidacy evaluation between 2017 and 2021. All patients ≥ 65 years old and those under 65 with any team member concern for frailty were referred for CGA, which included measurements of healthcare utilization, comorbidities, social support, short physical performance battery, Montreal Cognitive Assessment (MoCA), and Physical Frailty Phenotype (FPP), and estimate of surgical risk by the geriatrician.

**Results:**

Two hundred and thirty patients underwent baseline CGA evaluation; 58.7% (135) had high CGA (“Excellent” or “Good” rating for transplant candidacy) and 41.3% (95) had low CGA ratings (“Borderline,” “Fair,” or “Poor”). High CGA rating (OR 8.46; *p* < 0.05), greater number of CGA visits (OR 4.93; *p* = 0.05), younger age (OR 0.88; *p* < 0.05), higher MoCA scores (OR 1.17; *p* < 0.05), and high physical activity (OR 4.41; *p* < 0.05) were all associated with listing on transplant waitlist.

**Conclusions:**

The CGA is a useful, comprehensive tool to help select older adults for kidney transplantation. Further study is needed to better understand the predictive value of CGA in predicting post-operative outcomes.

**Supplementary Information:**

The online version contains supplementary material available at 10.1186/s12877-024-04734-7.

## Background

End-stage renal disease (ESRD) affects approximately 1,500 per million people in the United States, with increasing prevalence in patients aged 65 and older (50.4% as of 2019) [1, 2]. Kidney transplantation provides long-term benefits over dialysis, particularly survival, cost, and quality of life [3–5]. The American College of Surgeons National Surgical Quality Improvement Program and the American Geriatrics Society (2012) recommend evaluating older adults prior to surgery across several domains, including assessment of cognitive ability and capacity, screening for depression, functional status, history of falls, baseline frailty score, nutritional status, medication history and polypharmacy, family and social support system, treatment goals and expectations, risk for delirium, screening of substance abuse and dependences.

Frailty is a clinical syndrome characterized by decreased physiological reserve and increased vulnerability for poor health outcomes [6]. Measuring frailty helps assess surgical risk for pre-operative transplant patients [7, 8], since higher degrees of frailty predict adverse outcomes following kidney transplantation [9]. However, there are over sixty validated frailty assessment tools and many programs do not regularly assess frailty using a validated tool [10, 11].

Comprehensive geriatric assessment (CGA) is a broad term that alludes to a formal assessment, typically in older adults, of frailty and various health needs in multiple domains (i.e., cognitive, physical, social, functional) that is summarized into a plan for management [12]. At our institution, CGAs measured healthcare utilization, comorbidities, social support, Short Physical Performance Battery (SPPB), Montreal Cognitive Assessment (MoCA), and Physical Frailty Phenotype (PFP) in addition to modifiers of frailty (i.e., polypharmacy, dementia, disability, physical function, psychological health, polypharmacy, home service utilization, geriatric syndromes [13], and social, instrumental, and financial support) [14]. However, the impact of CGA and its components on the decision to list for kidney transplant in the older adult population has not been investigated. In this study, we determine the relationship between CGA and kidney transplant listing to provide a framework for comprehensively assessing risk associated with frailty during multidisciplinary discussion for kidney transplant listing.

## Methods

### Patient selection

**Fig. 1:**
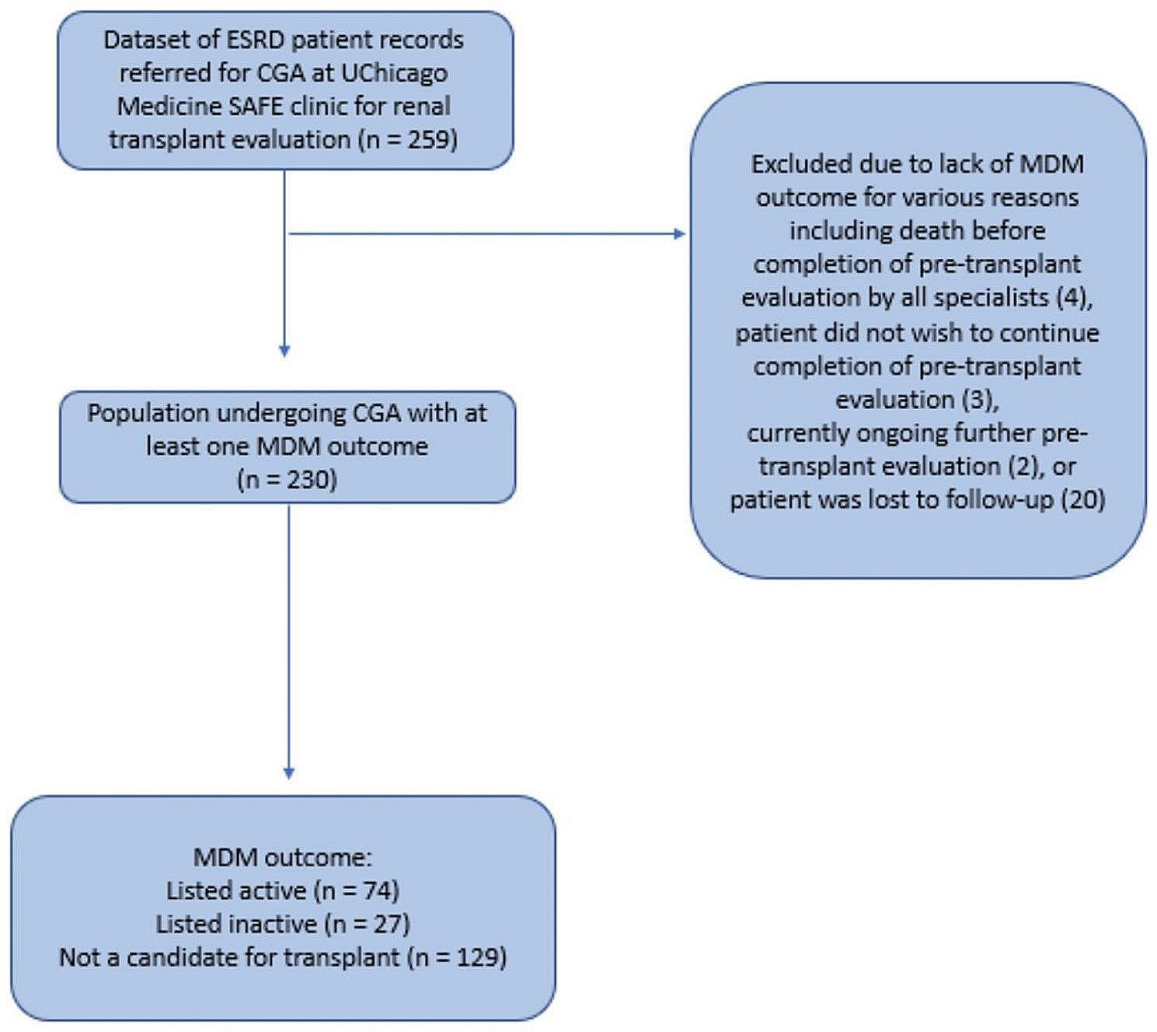
Patient selection criteria. The dataset of patients that underwent pre-renal transplant evaluation at our university consisted of 259 patients. Of those, 29 patients were excluded for a variety of reasons as they did not have an MDM outcome

A retrospective chart review of ESRD patients undergoing CGA during kidney transplant evaluation between 01/2017 and 07/2021 at the University of Chicago Medicine was performed (Fig. 1). Members of the transplant team referred all patients ≥ 65 years old or those under 65 with team member concern for presence of frailty or other geriatric syndromes to one geriatrician (M.M.) for CGA. Each CGA visit involved assessments of healthcare utilization, comorbidities, social support, SPPB, MoCA, PFP, and other validated tools relating to cognitive, physical, social, and functional domains of health (Supplemental Table S1). A decision support tool, [14] which was developed at our institution to standardize language involving estimate of risk from a geriatrics perspective, was utilized at each visit. At the end of each CGA visit, the geriatrician provided an overall CGA rating (ranging from “Excellent,” “Good,” “Borderline,” “Fair,” and “Poor”) of the patient’s candidacy for transplant. In our analysis, we grouped patients into those with a “high CGA rating” (overall CGA rating of “Excellent” or “Good”) and those with a “low CGA rating” (overall CGA rating of “Borderline,” “Fair,” or “Poor”) for comparison. The specifics of the CGA, as well as specific criteria used to determine CGA rating and clinician estimate of surgical risk are detailed in a previously published paper from our institution [14]. Abnormal findings during the CGA were managed on an individual basis, however, typical interventions are listed in Supplemental Table S2. Following CGA, patients were discussed in a multi-disciplinary meeting to decide if they should be listed for kidney transplant, deferred, or deemed ineligible for transplant. All members of the multi-disciplinary team were able to view results of the CGA evaluation. The study was approved by the University of Chicago Institutional Review Board (IRB21-1322).

A total of 29 patients did not have a final multi-disciplinary meeting (MDM) decision and were excluded from the study. The reasons for exclusion included: death before completion of pre-transplant evaluation by all specialists (*n* = 4), patient did not wish to complete pre-transplant evaluation (*n* = 3), currently ongoing further pre-transplant evaluation (*n* = 2), or patient was lost to follow-up (*n* = 20). No differences in CGA rating were noted in excluded patients.

### Data Collection

Chart review was performed to collect patient characteristics, intraoperative course, postoperative outcomes, CGA domains, geriatrician’s assessment of patient risk, and final MDM decision. Patient zip codes were used to approximate median incomes (using 2006–2010 data published by the University of Michigan Population Studies Center) [15] and social vulnerability indices (using 2018 data from Agency for Toxic Substances and Disease Registry) [16] of patients. Five factor modified frailty index (mFI-5) scores were calculated using data available in charts following previously published guidelines [17].

### Statistical analysis

Categorical variables were reported as frequencies and proportions; χ^2^ or Fischer’s Exact Test was used to calculate p-values between groups. Continuous variables were reported as means with standard deviations; an independent samples t-test was used to calculate p-values between groups. Binary logistic regression, which included clinically relevant variables and variables significant in univariate analysis, was used to calculate multivariable odds ratios (OR’s). Variables that crossed 0.5 in Pearson’s correlation coefficient with the outcome or were co-linear with other variables in the model were removed from the final multivariable logistic regression model. Breslow (generalized Wilcoxon) was used to determine difference in survival of patients following their latest CGA visit. Data were analyzed using SPSS statistical software (IBM SPSS statistics version 21).

## Results

### Characteristics of ESRD patients undergoing CGA and evaluation for kidney transplant

Table 1 shows characteristics of 230 patients who were evaluated. The mean and median follow-up times post-CGA were 13.6 and 10.5 months, respectively. A total of 135 patients (58.7%) had high CGA ratings for kidney transplant candidacy while 95 (41.3%) had low CGA ratings. Patients with high CGA ratings were older (69.3 ± 6.0 versus 67.4 ± 7.8) with higher average median income ($60,578 vs. $49,268), had lower rates of congestive heart failure, stroke, dementia, and coronary artery disease, but had a higher rate of peripheral vascular or arterial disease compared to patients with low CGA ratings (Table 1). Mean mFI-5 scores were higher in patients with low CGA ratings (Table 1). No differences were found between the two groups regarding sex, BMI, and social vulnerability index.

**Table 1:** Characteristics of ESRD patients undergoing pre-renal transplant CGA (*n* = 230)

	All *N* = 230 (100.0%)	High CGA rating *N* = 135 (58.7%)	Low CGA rating *N* = 95 (41.3%)	P-value^†^
Age in years– mean ± SD	68.5 ± 6.8	69.3 ± 6.0	67.4 ± 7.8	**0.043**
Age > 70 years– no. (%)	116 (50.4)	75 (55.6)	41 (43.2)	0.064
BMI in kg/m^2^– mean ± SD	28.9 ± 6.6	28.7 ± 5.8	29.1 ± 7.6	0.691
Female sex– no. (%)	108 (47.0)	60 (44.4)	48 (50.5)	0.363
Race^‡^– no. (%)				
Asian or Pacific Islander	15 (6.5)	9 (6.7)	6 (6.3)	0.915
Black	123 (53.5)	65 (48.1)	58 (61.1)	0.053
White	74 (32.2)	50 (37.0)	24 (25.3)	0.060
More than once race	8 (3.5)	6 (4.4)	2 (2.1)	0.340
Unknown	10 (4.3)	5 (3.7)	5 (5.3)	0.568
5-Factor Modified Frailty Index– mean ± SD^a^	2.16 ± 1.0	1.78 ± 0.9	2.69 ± 0.9	**< 0.001**
Comorbidities– no. (%)				
Hypertension	209 (90.9)	124 (91.9)	85 (89.5)	0.643
Diabetes	151 (65.7)	82 (60.7)	69 (72.6)	0.062
Cancer	32 (13.9)	19 (14.1)	13 (13.7)	0.933
Congestive Heart Failure	38 (16.5)	13 (9.6)	25 (26.3)	**< 0.001**
Arthritis	44 (19.1)	24 (17.8)	20 (21.1)	0.534
Stroke	31 (13.5)	12 (8.9)	19 (20.0)	**0.015**
Peripheral vascular or arterial disease	11 (4.8)	9 (6.7)	2 (2.1)	**< 0.001**
Dementia	4 (1.7)	0 (0.0)	4 (4.2)	**0.028**
Coronary artery disease or myocardial infarction	65 (28.3)	31 (23.0)	34 (35.8)	**0.033**
Delirium	2 (0.9)	0 (0.0)	2 (2.1)	0.170
Prior transplant– no. (%)				
Renal	17 (7.4)	12 (8.9)	5 (5.3)	0.443
Non-renal	3 (1.3)	2 (1.5)	1 (1.1)	1.000
Depression– no. (%)	22 (9.6)	9 (6.7)	13 (13.7)	0.110
Education– no. (%)				
Less than high school	30 (13.0)	16 (11.9)	14 (14.7)	0.522
High school	51 (22.8)	30 (22.2)	21 (22.1)	0.983
College	80 (34.8)	47 (34.8)	33 (34.7)	0.990
Graduate	24 (10.4)	18 (13.3)	6 (6.3)	0.087
Unknown	45 (19.6)	24 (17.8)	21 (22.1)	0.415
Median income– mean ± SD	$55,907 ± $4,258	$60,578 ± $52,785	$49,268 ± $19,253	**0.047**
Social Vulnerability Index^b^– mean ± SD	0.63 ± 0.3	0.60 ± 0.29	0.66 ± 0.28	0.102

^†^P-values calculated between the “Excellent”/”Good” and “Borderline”/”Fair”/”Poor” groups

^‡^No patients in the study were “Native American or Alaska Native.”

^a^5-factor Modified Frailty Index was scored from 0–5, with 5 being more frail

^b^Social vulnerability indices were acquired from CDC; higher score indicates greater vulnerability in region

### Relationship between CGA parameters, MDM decision, and overall CGA rating for kidney transplant candidacy

**Fig. 2:**
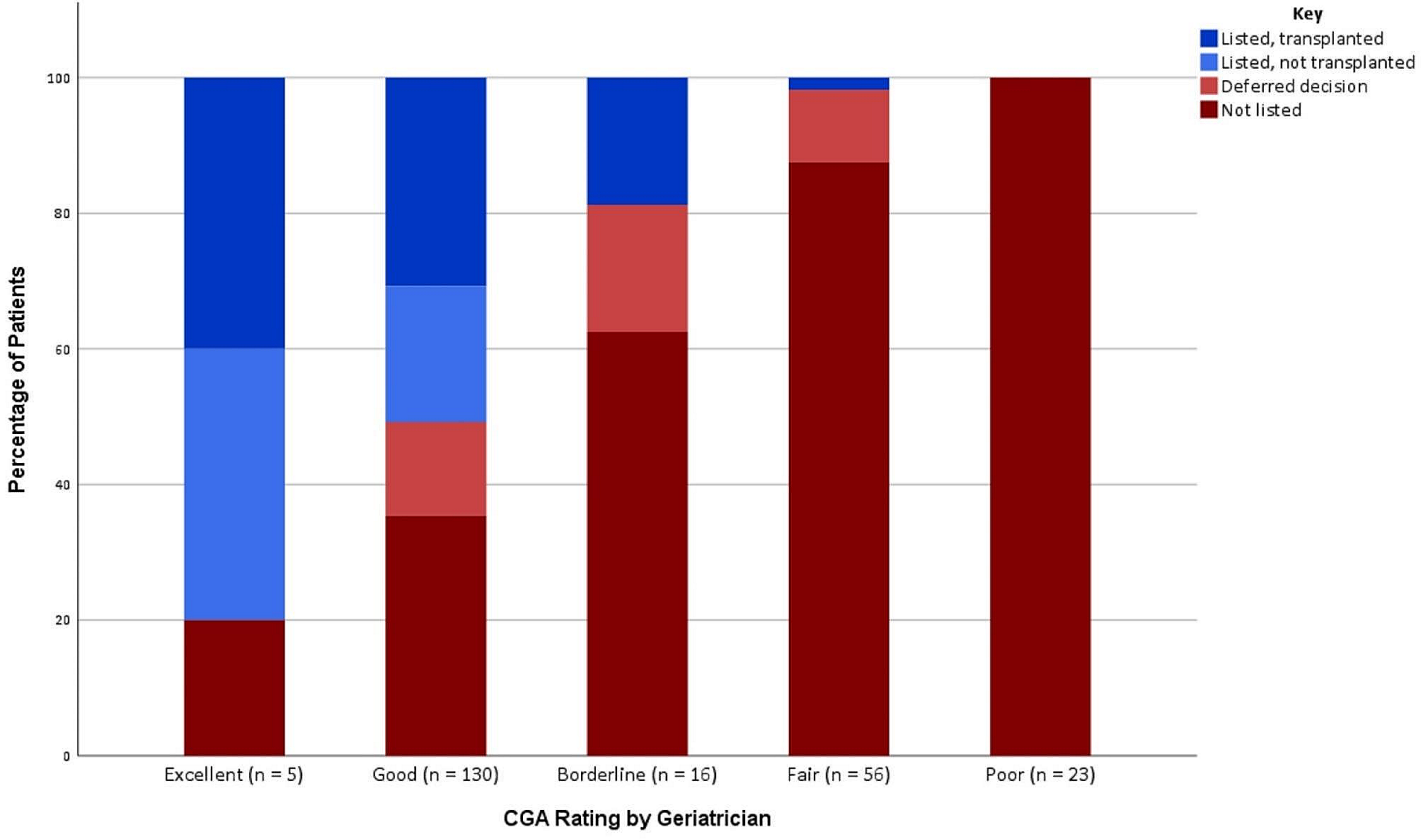
Post-CGA outcomes of patients at the conclusion of the study. The 100% stacked bar graph shows the outcomes, broken down by percentage, of all 230 patients that underwent pre-renal transplant CGA.

Table 2 shows the differences in CGA parameters among candidates with high versus low CGA ratings for kidney transplant candidacy. Patients with high CGA ratings were more likely to be independent (ADLs, iADLs), have a living will, not be on dialysis, have less polypharmacy, lower VES-13, lower PFP, lower PHQ-2, higher MoCA, and higher SPPB. Patients with low CGA ratings were more likely to have gait instability (two or more falls in last year and difficulty with balance), healthcare utilizations (at least one hospital admission, emergency room visit, or subacute rehab stay) in the last year, low physical activity, inadequate social support (as determined by geriatrician during visit), poorly controlled comorbidities, and an above average or higher geriatrician overall estimate of surgical risk. A total of 46 patients underwent transplantation (20%), with the majority (91.3%, 42/46) having high CGA ratings (Fig. 2). Four patients with low CGA ratings had improvements to their health status prior to being listed and subsequently transplanted (8.7%, 4/46).

**Table 2:** Patients with high CGA ratings by geriatrician and patients listed on transplant waitlist both have lower degrees of frailty compared to patients with low CGA ratings and those not listed, respectively. P-values, calculated through independent samples t-tests and Chi-squared or Fischer’s Exact tests, show the similarities and differences in parameters between the cohorts stratified by the geriatrician’s recommendation for transplant (CGA rating) and MDM decision

Assessed during CGA	All	CGA rating		P-value^†^	MDM decision		P-value^*^
	*N* = 230 (100.0%)	High *N* = 135 (58.7%)	Low *N* = 95 (41.3%)		Listed active *N* = 74 (32.2%)	Deferred or not a candidate *N* = 156 (67.8%)	
Number of CGA visits– mean ± SD	1.22 ± 0.5	1.24 ± 0.6	1.18 ± 0.5	0.375	1.36 ± 0.7	1.15 ± 0.5	**0.005**
Still driving– no. (%)	128 (55.7)	92 (68.1)	36 (37.9)	**< 0.001**	51 (68.9)	77 (49.4)	**0.005**
Two or more falls in last year– no. (%)	37 (16.1)	9 (6.7)	28 (29.5)	**< 0.001**	3 (4.1)	34 (21.8)	**< 0.001**
Difficulty with balance– no. (%)	85 (37.0)	29 (21.5)	56 (58.9)	**< 0.001**	19 (25.7)	66 (42.3)	**0.015**
Hospital admission in last year– no. (%)	115 (50.0)	54 (40.0)	61 (64.2)	**< 0.001**	27 (36.5)	88 (56.4)	**0.005**
Low physical activity– no. (%)	100 (43.5)	30 (22.2)	70 (73.7)	**< 0.001**	14 (18.9)	86 (55.8)	**< 0.001**
Surgical risk– no. (%)				**< 0.001**			**< 0.001**
Average	51 (22.2)	50 (37.0)	1 (1.1)		27 (36.5)	24 (15.4)	
Above average	116 (50.4)	85 (63.0)	31 (32.6)		47 (63.5)	69 (44.2)	
Significantly increased	56 (24.3)	0 (0.0)	56 (58.9)		0 (0.0)	56 (35.9)	
High	7 (3.0)	0 (0.0)	7 (7.4)		0 (0.0)	7 (4.5)	
Social Support				**< 0.001**			**0.005**
Adequate	212 (92.2)	132 (97.8)	80 (84.2)		73 (98.6)	139 (89.1)	
Inadequate	17 (7.4)	2 (1.5)	15 (15.8)		0 (0.0)	17 (10.9)	
Unknown	1 (0.4)	0 (0.0)	1 (0.7)		1 (1.4)	0 (0.0)	
Control of Comorbidities^‡^				**< 0.001**			**< 0.001**
None or well controlled	5 (2.2)	4 (3.0)	1 (1.1)		3 (4.1)	2 (1.3)	
Generally well controlled	166 (72.2)	125 (92.6)	41 (43.2)		70 (94.6)	96 (61.5)	
Poorly controlled	49 (21.3)	4 (3.0)	45 (47.4)		1 (1.4)	48 (30.8)	
Unable to be determined	10 (4.3)	2 (1.5)	8 (8.4)		0 (0.0)	10 (6.4)	
ADLs^a^, assistance	0.2 ± 0.7	0.5 ± 0.0	1.0 ± 0.2	**< 0.001**	0.0 ± 0.3	0.3 ± 0.8	**< 0.001**
iADLs^b^– mean ± SD							
Combined	1.9 ± 2.3	1.0 ± 1.7	3.9 ± 2.5	**< 0.001**	0.9 ± 1.5	2.8 ± 2.7	**< 0.001**
Dependency	0.6 ± 0.5	0.1 ± 0.4	0.6 ± 1.3	**0.001**	0.2 ± 0.5	0.4 ± 1.1	**< 0.001**
Assistance	0.3 ± 0.9	0.9 ± 1.6	3.3 ± 2.4	**< 0.001**	0.8 ± 1.4	2.4 ± 2.4	**< 0.001**
VES-13^c^– mean ± SD	2.5 ± 2.6	1.1 ± 1.7	4.5 ± 2.3	**< 0.001**	1.0 ± 1.6	3.2 ± 2.7	**< 0.001**
Medications– mean ± SD	10.2 ± 4.7	9.4 ± 4.5	11.4 ± 4.8	**0.003**	10.1 ± 4.7	10.2 ± 4.7	0.661
History of Tobacco use– no. (%)	91 (39.6)	47 (34.8)	44 (46.3)	0.079	19 (25.7)	72 (46.2)	**0.003**
ETOH use– no. (%)	24 (10.4)	18 (13.3)	6 (6.3)	0.229	6 (8.1)	15 (9.6)	0.711
Health care power of attorney– no. (%)	69 (30.0)	41 (30.4)	29 (21.5)	0.885	24 (32.4)	45 (28.8)	0.536
Living will– no. (%)	15 (6.5)	13 (9.6)	2 (2.1)	**0.028**	7 (9.5)	8 (5.1)	0.259
Use of healthcare facilities in last year– no. (%)							
ER visit	87 (37.8)	38 (28.1)	49 (51.6)	**< 0.001**	22 (29.7)	65 (41.7)	0.081
Long term care facility	1 (0.4)	0 (0.0)	1 (1.1)	0.413	0 (0.0)	1 (0.6)	1.000
Subacute rehab	20 (8.7)	4 (3.0)	16 (16.8)	**< 0.001**	4 (5.4)	16 (10.3)	0.223
Acute rehab	1 (0.4)	1 (0.7)	0 (0.0)	1.00	1 (1.4)	0 (0.0)	0.322
Months on dialysis^§^– mean ± SD	40.5 ± 42.2	39.1 ± 43.6	42.4 ± 40.4	0.607	44.8 ± 48.6	38.5 ± 38.8	0.351
Not on dialysis– no. (%)	36 (15.7)	26 (19.3)	8 (8.4)	**0.023**	15 (20.3)	19 (12.2)	1.000
MoCA^d^– mean ± SD	23.9 ± 4.0	24.9 ± 3.5	22.5 ± 4.2	**< 0.001**	25.5 ± 3.2	23.1 ± 4.1	0.061
Mini cog^e^– mean ± SD	3.6 ± 1.3	3.8 ± 1.3	3.2 ± 1.2	0.074	4.0 ± 1.3	3.4 ± 1.3	0.765
SPPB^f^– mean ± SD							
Balance	2.8 ± 1.4	3.5 ± 0.9	1.9 ± 1.4	**< 0.001**	3.3 ± 1.1	2.6 ± 1.4	**0.003**
Gait	3.2 ± 1.2	3.9 ± 0.4	2.3 ± 1.5	**< 0.001**	3.8 ± 0.5	3.0 ± 1.4	**< 0.001**
Chair stands	1.5 ± 1.3	2.0 ± 1.3	0.7 ± 1.0	**< 0.001**	2.1 ± 1.4	1.2 ± 1.2	0.119
Total	7.5 ± 3.3	9.3 ± 1.9	4.9 ± 3.1	**< 0.001**	9.2 ± 2.2	6.7 ± 3.4	**< 0.001**
Physical Frailty Phenotype^g^– mean ± SD	1.6 ± 1.3	0.8 ± 0.7	2.7 ± 1.2	**< 0.001**	0.9 ± 0.9	1.9 ± 1.4	**< 0.001**
PHQ-2^h^– mean ± SD	0.3 ± 0.9	0.2 ± 0.7	0.5 ± 1.2	**0.012**	0.2 ± 0.5	0.4 ± 1.1	**0.012**
CGA rating of “Excellent” or “Good”	135 (58.7)	--	--	--	70 (94.6)	65 (41.7)	**< 0.001**

^†^P-values calculated between the “Excellent”/”Good” and “Borderline”/”Fair”/”Poor” groups

*P-values calculated between the “Listed active” and “Deferred or not a candidate” groups

^§^Months on dialysis calculated as difference between initial date of starting dialysis in EMR and latest CGA visit

^‡^Control of comorbidities of patients was determined by geriatrician during the CGA.

^a^Activities of daily living (ADLs), including dressing, bathing, feeding, toileting, transferring, and continence. No patients had dependency in ADLs.

^b^Instrumental activities of daily living (iADLs), including driving, medications, cooking, cleaning, finances, telephone use, shopping, and laundry, were summated to produce a combined and separated score for assistance and dependence

^c^Vulnerable Elders Survey (VES-13) score ranges from 0–10. A score of 2 or less is considered normal. 6 or higher indicates high risk for postoperative complications

^d^Montreal Cognitive Assessment (MoCA) score ranges from 0–30. A score of 25 or lower is considered abnormal

^e^The Mini-Cog© score ranges from 0–5. A score of 1 or 2 is indicative of possible dementia or cognitive impairment

^f^The short physical performance battery (SPPB) test is a lower extremity physical function test scored from 0–12 (4 maximum points from Balance, Chair Stands, and Gait). A score of 7–9 suggests mild impairment while 0–6 suggests severe impairment

^g^Physical Frailty Phenotype (PFP) test is scored as 0 = Not frail, 1–2 = Pre-frail, 3–5 = Frail

^h^Patient Health Questionnaire-2 (PHQ-2) of 3 or higher is cutoff for possible Major Depressive Disorder

Table 2 also shows the differences in CGA parameters among candidates listed versus not listed for kidney transplant by MDM decision. Compared to patients listed on the transplant waitlist, those not listed were more likely to have gait instability (2 or more falls in the last year and have difficulty with balance), a history of tobacco use, at least one hospital admission in the last year, low physical activity, poorly controlled comorbidities, and above average or greater surgical risk estimated by geriatrician. Patients listed were more likely to be independent (lower mean ADL assistance, and lower mean iADL dependency and assistance) and have lower VES-13, lower PFP, lower PHQ-2, and higher SPPB scores. All parameters showing a difference between listed versus not listed patients were considered for logistic regression in multivariable analysis (Table 3).

**Table 3:** Multivariable logistic regression showing variables predicting listing on renal transplant waitlist. Variables that were significant predictors for listing on transplant waitlist, as well as clinically significant variables, were considered in the model. Variables were removed from the model if their Pearson’s correlation coefficient was 0.5 or higher or if they exhibited collinearity with other variables in the model

Category	Multivariable odds ratio (95% CI)	P-value
CGA Rating by Geriatrician		
High (“Excellent” or “Good”)	8.46 (1.37–52.40)	**0.022**
Low (“Borderline,” “Fair,” or “Poor”)	Reference	N/A
Number of CGA visits	4.93 (1.61–15.11)	**0.005**
Age at CGA visit	0.88 (0.79–0.98)	**0.024**
MoCA score	1.17 (1.01–1.35)	**0.032**
History of tobacco use	0.39 (0.14–1.09)	0.072
High physical activity	4.41 (1.09–17.78)	**0.037**
Control of comorbidities		
Generally well controlled, well controlled, or none	6.84 (0.59–79.03)	0.123
Poorly controlled	Reference	N/A
Two or more falls in last year	0.00 (0.00–0.00)	0.998

### Multivariable analysis of CGA parameters predicting patient listing for kidney transplant

Multivariable logistic regression was performed after including CGA rating by geriatrician, number of CGA visits, age at CGA, MoCA score, history of tobacco use, high physical activity, control of comorbidities, and fall history in the model. CGA rating by geriatrician (OR 8.46 95% CI 1.37–52.40), number of CGA visits (OR 4.93 95% CI 1.61–15.11), age at CGA visit (treated as continuous variable; OR 0.88 95% CI 0.79–0.98), MoCA score (OR 1.17 95% CI 1.01–1.35), and high physical activity (OR 4.41 95% CI 1.09–17.78) were all significant predictors for listing for kidney transplant (Table 3).

### Survival

Patients had mean and median follow-up times of 13.6 and 10.5 months following the CGA, respectively. In total, there were 22 deaths in the study (9.6%). A Breslow (generalized Wilcoxon) test showed that there was a difference in survival between patients with high versus low CGA ratings with those in the former group surviving longer (*p* = 0.038).

## Discussion

Kidney transplantation for patients with ESRD can lead to improved quality of life and longevity relative to patients who remain on dialysis [18, 19]. There is significant variation in pre-operative assessment of older adults across various transplant centers [20]. Given the association of frailty with poor outcomes following kidney transplantation, [3, 7, 8, 19, 21] the CGA is a promising tool as it comprehensively assesses frailty through several validated metrics and fulfills many of the recommended items for pre-operative evaluation of older adults by the American College of Surgeons National Surgical Quality Improvement Program and the American Geriatrics Society [22].

CGAs have existed for a few decades, and take more holistic approaches to evaluating the health of older adults, encompassing formal measurements of frailty and domains of health in addition to creating a plan for management [23]. To our knowledge, this is the first study assessing the impact of CGA on kidney transplant decision-making and listing in ESRD patients. Our study differs from other studies utilizing CGA in that the geriatrician used a clinical decision support tool to stratify candidates based on surgical risk (through classifying candidates as “Excellent,” “Good,” “Borderline,” “Fair,” or “Poor”) [23]. Although geriatrician recommendations could be simplified to “recommend” or “not recommend” (and in some cases, an intermediate zone), we believed a more granular approach with five categories was more appropriate given the comprehensive nature of the CGA. We found that the geriatrician’s CGA rating, the number of CGA appointments, age, MoCA score, and high physical activity were all significantly associated with MDM decision to list patients for kidney transplant in our multivariable logistic regression model. It should be noted that the geriatrician actively participated in the MDM and incorporated the results of the CGA into the decision-making process for listing. In addition, we found that several vulnerability domains (healthcare utilization, comorbidities, SPPB, social support, and PFP) differed among both patients listed versus not listed and patients given high CGA ratings versus low CGA ratings. The results of our study also show that patients with high CGA ratings had greater survival following their latest CGA. However, it should be noted that the mean follow-up time was only 13.6 months, and follow-up bias may have occurred given that patients had their CGA visits at different time points during the study period.

Although clinicians can use different frailty assessment tools to assess frailty in ESRD patients being evaluated for kidney transplant, there tends to be only moderate or fair agreement between them [24]. Haugen et al. showed that more frail patients, measured by PFP, are less likely to be placed on the transplant waitlist, [25] while Harhay et al. found that frail patients on the waitlist have higher mortality [26]. Despite frailty increasing risk for post-transplant adverse outcomes, transplantation in frail ESRD patients can improve quality of life [27]. Patients who are kidney transplant candidates have also expressed discomfort in clinical use of single constructs, such as frailty or cognitive scores, to deny potential candidates the ability to acquire a transplant [28]. Shrestha et al. surveyed expert clinicians who care for ESRD patients and found that they believed that frailty and cognitive scores should be evaluated in the context of other factors, such as frailty reversibility [29]. Whereas more concise measurements of frailty may focus on certain aspect of one’s health (i.e., cognitive and functional status or physical function), the CGA allows for the geriatrician to holistically evaluate a patient and make comprehensive recommendations to manage aspects of frailty and health that would establish surgical risk, inform eligibility, implement pre-operative interventions that may help reduce surgical risk and frailty and prepare for peri- and post-operative care. Although there is controversy regarding the reversibility of frailty, aspects of frailty (such as physical frailty) may be managed through methods such as adequate protein-calorie consumption, exercise, or reduction of polypharmacy [21]. In our study, we found that more CGA visits were associated with a greater likelihood of transplantation. Generally, patients who had absolute contraindications (i.e., history of renal cell carcinoma or significant calcification of vasculature) did not have a path back for transplant reevaluation. However, if the reason for not listing was related to a modifiable risk factor (i.e., high BMI or poor physical function), patients were provided recommendations prior to a repeat CGA the following year. Patients who were listed or were borderline candidates for transplant (usually with deferred waitlist decision) were seen annually for follow-up visits. Repeated assessments at multiple time points is a strength of the CGA as it allows for monitoring of improvements of health status and optimization of outcomes in patients who are borderline candidates for transplantation. It should be noted that patients at the time of their first CGA visit had variable amounts of time on dialysis and that chronic dialysis can affect one’s physical function (e.g., cardiovascular health), and in turn, frailty [30].

Though the CGA allows for a more multidimensional evaluation of older adults, it comes with a few drawbacks. Firstly, it is time- and resource-intensive given the multiple assessments that are involved. Secondly, not all hospital systems may have the resources and facilities to perform CGAs. However, with an aging population and shift of burden of disease from acute to chronic in the United States, a more holistic evaluation of frailty and key measures of health is important for equitable selection of transplant candidates.

## Conclusion

Our study is a single-center retrospective observational study which demonstrated the association of CGA with kidney transplant listing. All 230 patients were seen by the same geriatrician, but multiple transplant clinicians were involved in the final MDM decision to list. A prospective trial with a larger cohort of patients and longitudinal follow-up may shed light on how CGA may predict post-transplant outcomes and how serial CGAs could help manage frailty and improve chances for kidney transplant listing.

### Limitations

Our study was limited by its retrospective nature and short follow up. Although CGA ratings were based on a published standardized decision support tool, a sole geriatrician performed all the CGAs in the study, which may have had single evaluator bias. At our institution, we do not have a defined, objective criteria to refer patients for pre-operative kidney transplant evaluation in patients under the age of 65. In the study, five patients were under 50 and 48 were under 65. In addition, our survival analysis had follow-up bias since patients had their CGA visits at different points.

### Electronic supplementary material

Below is the link to the electronic supplementary material.

Supplementary Material 1

Supplementary Material 2

## Data Availability

Although collected patient data was de-identified prior to analysis, data sharing is not available as the risk for breaches of confidentiality must be minimized by limiting data access. A request for data should be sent to the corresponding author (JP).

## Abbreviations

CGA: Comprehensive Geriatric Assessment
ESRD: End-Stage Renal Disease
ADL: Activities of Daily Living
iADL: Instrumental Activities of Daily Living
MoCA: Montreal Cognitive Assessment
VES-13: Vulnerable Elders Survey 13
PFP: Physical Frailty Phenotype
SPPB: Short Physical Performance Battery
mFI-5: Five Factor Modified Frailty Index
MDM: Multi-Disciplinary Meeting
