# Supplementary material for: Comprehensive geriatric assessment predicts listing for kidney transplant in patients with end-stage renal disease: a retrospective cohort study
Source: BMC Geriatr. 2024 Feb 13;24:148. doi: 10.1186/s12877-024-04734-7 (PMC10865555; doi:10.1186/s12877-024-04734-7)
Supplement: Supplementary file 1 — Supplementary Material 1 [file 12877_2024_4734_MOESM1_ESM.docx]

**Supplemental Table S1.** Different components of the CGA

| CGA Component | Description |
| --- | --- |
| Activities of daily living (ADL)^1^ | Dependency or required assistance in various ADLs, including dressing, bathing, feeding, toileting, transferring, and continence were noted |
| Instrumental activities of daily living (iADL)^1^ | Dependency or required assistance in various iADLs, including driving, medications, cooking, cleaning, finances, telephone use, shopping, and laundry were noted |
| Vulnerable Elders Survey (VES-13)^2^ | The VES-13 score ranges from 0-10. A score of 2 or less is considered normal. 6 or higher indicates high risk for postoperative complications. |
| Montreal Cognitive Assessment (MoCA)^3^ | The MoCA score ranges from 0-30. A score of 25 or lower indicates cognitive impairment. |
| Short physical performance battery (SPPB)^4^ | The SPPB test is a lower extremity physical function test scored from 0-12. There are 3 components (balance, chair stands, and gait) that each have a maximum of 4 attainable points. A score of 7-9 suggests mild physical impairment while a score of 6 or less suggests severe impairment. |
| Physical Frailty Phenotype (PFP)^5^ | The PFP test is scored out 5 points and includes five criteria: unintentional weight loss, self-reported exhaustion, weakness (grip strength), slow walking speed, and low physical activity. A score of 0 indicates no frailty while 1-2 and 3-5 indicate pre-frailty and frailty, respectively. |
| Patient Health Questionnaire-2 (PHQ-2)^6^ | The PHQ-2 depression screen is a 2-item questionnaire that asks about the regularity of anhedonia and depressed mood. It is scored from 0-6, with 3 or higher as a cutoff for Major Depressive Disorder. |
| Other assessments | Other components of the CGA included marital status, education, history of alcohol and tobacco use, comorbidities, prior transplant history, BMI, use of social services, driving status, gait instability (fall history in last year and difficulty with balance), living environment, healthcare utilization in last year (hospitalization, ER visit, subacute rehab, acute rehab, long-term care, palliative care, and hospice), polypharmacy, control of comorbidities (poorly or well controlled), physical activity (high, low, or undetermined, based on patient history), social support (adequate or inadequate), |

1. Katz S. Assessing self-maintenance: activities of daily living, mobility, and instrumental activities of daily living. *Journal of the American Geriatrics Society.* 1983.

2. Saliba D, Elliott M, Rubenstein LZ, et al. The Vulnerable Elders Survey: a tool for identifying vulnerable older people in the community. *Journal of the American Geriatrics Society.* 2001;49(12):1691-1699.

3. Nasreddine ZS, Phillips NA, Bédirian V, et al. The Montreal Cognitive Assessment, MoCA: a brief screening tool for mild cognitive impairment. *Journal of the American Geriatrics Society.* 2005;53(4):695-699.

4. Guralnik JM, Simonsick EM, Ferrucci L, et al. A short physical performance battery assessing lower extremity function: association with self-reported disability and prediction of mortality and nursing home admission. *Journal of gerontology.* 1994;49(2):M85-M94.

5. Fried LP, Tangen CM, Walston J, et al. Frailty in older adults: evidence for a phenotype. *The Journals of Gerontology Series A: Biological Sciences and Medical Sciences.* 2001;56(3):M146-M157.

6. Kroenke K, Spitzer RL, Williams JB. The Patient Health Questionnaire-2: validity of a two-item depression screener. *Medical care.* 2003:1284-1292.
