# Supplementary material for: Comprehensive geriatric assessment predicts listing for kidney transplant in patients with end-stage renal disease: a retrospective cohort study
Source: BMC Geriatr. 2024 Feb 13;24:148. doi: 10.1186/s12877-024-04734-7 (PMC10865555; doi:10.1186/s12877-024-04734-7)
Supplement: Supplementary file 2 — Supplementary Material 2 [file 12877_2024_4734_MOESM2_ESM.docx]

**Supplemental Table S2**. A list of typical interventions by the geriatrician during the CGA.

| CGA Finding | Recommendations |
| --- | --- |
| Weak grip strength or low SPPB score | Home exercise program |
| Frail or Pre-frail based on PFP | Recommend physical activity; referral to PT; referral to nutritionist; control of co-morbidities |
| PHQ-2 score of 3 or higher | Consider referral or initiating treatment (psychotherapy or pharmacotherapy) for depression |
| Polypharmacy | Review medications and side effects; discuss safer alternatives if medications on Beer’s list; assess medication compliance; medication reconciliation; medication education |
| Low BMI or weight loss | Referral to nutritionist; nutrition recommendations; strategies for weight maintenance; lab evaluation (TSH); recommend small and frequent meals; liberalize diet as much as possible, add spice to food, eat desired foods; eat high calorie foods; eat with other people |
| Low MoCA score | Bring caregiver or family member to appointments; discuss memory tools/aids and strategies; Labs (TSH, Vitamin B12, CMP, CBC) to rule out reversible causes of cognitive impairment, encourage socialization, physical activity, and healthy diet |
| No advanced directive on file | Recommend completion |
| Limited social support | Recommend finding social support as well as backup person |
| History of falls | Assess for causes (neurological, vision, gait/strength/balance, cardiovascular, check and replete vitamin D if not replete, footwear, environmental); referral to PT for strength and balance training; referral to occupational therapy for assessment of home safety; life alert button; durable medical equipment |
| History of delirium | Postoperative geriatrics consult for prevention; assessment of current medications to reduce risk |
| History of opioid addiction | Advanced planning for postoperative pain management |

Abbreviations: Short physical performance battery (SPPB); Physical Frailty Phenotype (PFP); Physical Therapy (PT); Thyroid stimulating hormone (TSH); Montreal Cognitive Assessment (MoCA); Complete metabolic panel (CMP); Complete blood count (CBC).
